# Lecanemab Subcutaneous Formulation for Maintenance Dosing in Early Alzheimer’s Disease (AD)

**DOI:** 10.1002/alz70859_104693

**Published:** 2025-12-25

**Authors:** Larisa Reyderman, Natasha Penner, Pratik Bhagunde, David Li, Shobha Dhadda, Steven Hersch, Michael C. Irizarry, Lynn D Kramer

**Affiliations:** ^1^ Eisai Inc., Nutley, NJ USA

## Abstract

**Background:**

Lecanemab is a humanized IgG1 monoclonal antibody with high affinity to Aβ soluble protofibrils. In two clinical studies (phase 2 and phase 3 Clarity AD) in early AD, lecanemab substantially reduced amyloid PET and slowed clinical decline on multiple measures of cognition and function, including CDR‐SB at 18 months. Herein, we present the rationale for continued lecanemab dosing with a weekly 360 mg subcutaneous formulation beyond the initial 18‐month treatment, utilizing semi‐mechanistic models and simulations correlating lecanemab exposure with amyloid PET, plasma biomarkers, and clinical outcomes.

**Methods:**

Models describing the relationship between serum lecanemab exposure, amyloid PET, and CDR‐SB have been previously described. Individual post‐hoc model estimates for pharmacokinetics, PET, and CDR parameters were obtained from Clarity AD subjects who received lecanemab and were used to simulate change in amyloid PET, CDR‐SB, and biomarkers (Ab42/40 ratio, ptau181, and GFAP) over 4 years. Simulations were conducted to evaluate the effect of lecanemab after continuous every 2‐week treatment or after transitioning to 360 mg subcutaneous weekly dosing at 18 months.

**Results:**

Models included data for 110 months from the lecanemab phase 2 study and 54 months of continuous lecanemab biweekly dosing from Clarity AD. Continued lecanemab treatment with 360 mg subcutaneous dosing resulted in a similar additional reduction of amyloid PET over 4 years of treatment as continued intravenous lecanemab. There were no differences in clinical outcomes (e.g. CDR‐SB) between continued biweekly intravenous doses and 360 mg subcutaneous maintenance dose regimens. The 360 mg subcutaneous formulation weekly dosing maintains plasma biomarker (Aβ42/40, GFAP, p‐tau181) at levels consistent with inhibition of AD pathology and neuroinflammation. Similar effects were observed across all assessments.

**Conclusions:**

Maintenance subcutaneous lecanemab dosing will reduce the burden for subjects and their caregivers associated with lecanemab administration while still maintaining the efficacy observed with lecanemab biweekly treatment. Modeling indicates that maintenance therapy with subcutaneous lecanemab prevents biomarker re‐accumulation and has no significant meaningful impact on amyloid or meaningful impact on disease progression compared to the intravenous regimen. A subcutaneous formulation has the potential to improve the lecanemab safety profile with a more patient friendly and convenient route of administration.

